# PrP^C^ Undergoes Basal to Apical Transcytosis in Polarized Epithelial MDCK Cells

**DOI:** 10.1371/journal.pone.0157991

**Published:** 2016-07-07

**Authors:** Alexander Arkhipenko, Sylvie Syan, Guiliana Soraya Victoria, Stéphanie Lebreton, Chiara Zurzolo

**Affiliations:** Unité de Trafic Membranaire et Pathogénèse, Institut Pasteur, 25-28 rue du docteur Roux, 75015, Paris, France; Deutsches Zentrum für Neurodegenerative Erkrankungen e.V., GERMANY

## Abstract

The Prion Protein (PrP) is an ubiquitously expressed glycosylated membrane protein attached to the external leaflet of the plasma membrane via a glycosylphosphatidylinositol anchor (GPI). While the misfolded PrP^Sc^ scrapie isoform is the infectious agent of prion disease, the cellular isoform (PrP^C^) is an enigmatic protein with unclear function. Of interest, PrP localization in polarized MDCK cells is controversial and its mechanism of trafficking is not clear. Here we investigated PrP traffic in MDCK cells polarized on filters and in three-dimensional MDCK cysts, a more physiological model of polarized epithelia. We found that, unlike other GPI-anchored proteins (GPI-APs), PrP undergoes basolateral-to-apical transcytosis in fully polarized MDCK cells. Following this event full-length PrP and its cleavage fragments are segregated in different domains of the plasma membrane in polarized cells in both 2D and 3D cultures.

## Introduction

The cellular isoform of the prion protein (PrP^C^) is a glycosylphosphatidylinositol-anchored protein (GPI-AP) ubiquitously expressed in different tissues, with high levels in the nervous and lymphoid tissues, and lower levels in muscles, heart, digestive tract and skin [1]. The physiological function of PrP^C^ is still elusive [2]. Prion protein has received considerable attention due to its central role in the development of Transmissible Spongiform Encephalopathies (TSEs), in animals and humans [1,3]. In these neurodegenerative disorders, PrP^C^ converts into a pathological conformation, called PrP^Sc^ (where Sc stands for Scrapie), which results in considerable toxicity to cells of the central nervous system, with neurons being, progressively, the most damaged. Additionally, several studies have previously reported that the alteration of intracellular trafficking of PrP affects the conversion of PrP^C^ to PrP^Sc^, and therefore PrP physiological function [4–6]. It has also been shown that in prion-infected cells, post-Golgi transport of PrP^C^ and other proteins are impaired, thereby possibly contributing to pathophysiology [7]. PrP at steady state undergoes post-translational processing generating N-ter and C-ter fragments [8–11]. For instance, in the sciatic nerve PrP cleavage fragment C1 is enriched in the axonal membrane where it is necessary to maintain surrounding myelin [12]. Different PrP cleavage fragments such as C1, C2, N1 and N2 have been shown to trigger different cell responses and to be of importance in the prion disease pathogenesis [6,10,13–15]. Thus, understanding the trafficking, the processing and degradation of PrP is of fundamental importance in order to unravel the mechanism of PrP^Sc^ mediated pathogenesis, its spreading and cytotoxicity. Neurons are a group of highly polarized cells with axons and dendrites exhibiting distinct structures and functions. Cell polarity is characterized by the development of asymmetry in the plasma membrane resulting from vectorial transport of proteins and lipids to create plasma membrane domains. The mechanisms of polarity establishment and plasma membrane domain biogenesis have been most extensively studied in epithelial cell models, thus providing a solid base upon which trafficking studies of individual proteins like PrP can be built in this context. Furthermore epithelial cells and neurons share common features regarding the mechanism of protein sorting [16,17]. It was proposed in 1990 that the mechanisms of protein sorting of viral proteins such as VSV and HA are similar in both Madin-Darby canine kidney (MDCK) and neurons [18–20]. Additionally, our current understanding of the sorting and trafficking of GPI-APs, including PrP, results mostly from studies performed in epithelial cells with a prevalence in MDCK cells [21–27].

We therefore focused on polarized PrP trafficking in MDCK cells as a relevant model in which to dissect trafficking and sorting mechanisms of PrP. Additionally, previous work addressing this issue [28–31] has also been carried out in MDCK cells stably transfected with the human or mouse PrP cDNA. Several studies from our group have also elucidated the trafficking of GPI-APs in MDCK, including the exceptional PrP [23–27, 29].

Pioneering work demonstrated that GPI-APs localize on the apical membrane in MDCK cells [21,22,32] as well as in other epithelial cell lines [25,32]. It was shown that GPI-APs which are preferentially sorted from the trans-Golgi network (TGN) to the apical surface follow a direct route from the Golgi apparatus to the plasma membrane [33]. Later work from our and other laboratories has unraveled the mechanism of apical sorting of GPI-APs, demonstrating that both association of GPI-APs with detergent resistant membranes (DRMs) and cholesterol dependent clustering in high molecular weight (HMW) complexes in the Golgi are necessary for this process [24,34–36].

The one exception to direct apical sorting of native GPI-APs in MDCK cells is represented by the prion protein. In 2002, work from our laboratory showed that mouse PrP localizes on the basolateral membrane of fully polarized MDCK cells [29]. Several groups later addressed the distribution and intracellular trafficking of PrP in polarized MDCK cells [28,30,31,37] but the data did not reach a clear resolution. While De Keukeleire and collaborators [28] as well as Christensen and Harris [37] found PrP at the apical membrane of MDCK cells, Uelhoff and colleagues [31] confirmed our findings showing a basolateral localization of PrP. The reason for these contradictory results has never been explained. The two major differences in these studies are in the PrP sequence and the anti-PrP antibodies used. While De Keukeleire worked with human PrP, the other studies employed mouse PrP. In addition, Christensen and Harris used the C-terminal PrP antibody SA65, while our laboratory used the N-terminal antibody SAF32. Uelhoff and colleagues introduced a 3F4 tag into the N terminal region of mouse PrP and used a 3F4 antibody, thereby also recognizing the region of the α-cleavage (aa 108–111 in mouse PrP). Of note, none of these publications took into account the posttranslational proteolytic processing of PrP. Because PrP at steady state undergoes extensive proteolytic modification, called α-cleavage [8–11], differential α-cleavage could also explain the contradictory localization of PrP in polarized MDCK cells.

In this work, we addressed PrP trafficking in MDCK cells, taking into account its proteolytic processing. Furthermore, we used MDCK cells polarized on filters and a three-dimensional (3D) culture system of MDCK cells cysts embedded in Matrigel^™^. 3D MDCK cysts recapitulate numerous features of epithelial tissues *in vivo* [38,39] and therefore provide a good model to study polarized protein trafficking under physiological conditions. We report here that full-length PrP and its cleavage fragments are segregated in different domains of the plasma membrane in polarized cells in both 2D and 3D cultures and that the ratio of the C-terminal fragment to PrP full-length increases upon MDCK polarization. We found that unlike other GPI-APs, PrP undergoes basolateral-to-apical transcytosis in fully polarized MDCK cells.

This study not only reconciles and explains the different findings in the previous literature but also provides a better picture of PrP trafficking and processing, which has been shown to have major implications for its role in prion disease [4,6,40–42].

## Material and Methods

### Reagents and antibodies

Cell culture media were purchased from Sigma-Aldrich (St. Louis, MO). Antibodies were purchased as follows: anti-actin, polyclonal α-GFP and monoclonal α-GFP from Invitrogen (Eugene, OR), SAF32 and SHA31 from Bertin pharma, France. Matrigel^™^ was purchased from Corning (France), Phalloidine-Alexa647 from Thermo; all other reagents were purchased from Sigma-Aldrich. Cycloheximide (CHX) was purchased from Millipore (Billerica, MA, USA).

### Cell culture

MDCK cells were grown in DMEM containing 5% fetal bovine serum. MDCK cells stably expressing mouse PrP has been described previously [29]. Cells were grown on coverslips for 24 hours (non-polarized condition) or plated for 5 days on transwell filters to obtain fully polarized cells. 3D cyst formation was performed as described in [43]. Briefly, 8 well chambers and pipette tips were cooled down, chambers were coated with 15 μl Matrigel^™^ freshly melted on ice. The gel coating was solidified for 15 min at 37°C. A low-density cell suspension (20 000 cells /ml of media) was prepared in DMEM containing Penicillin/streptomycin 5ml (100x), 5% FBS and 2% Matrigel^™^. 200 μl of cell suspension was plated in each well. Media containing 2% Matrigel^™^ was changed every 3 days.

### Deglycosylation assay and Western Blotting

For deglycosylation, cells were lysed in NP-40 lysis buffer (25mM Tris pH 7.5, 150 mM NaCl, 1% NP-40), and protein concentration in the cell lysate was quantified with Pierce BCA Protein Assay Kit (Thermo scientific). 40 μg of protein was treated with 50 units of PNG^ase^ (New England Biolabs, MA) at 37°C for 1h with agitation. Samples were mixed with SDS-loading dye and run on a 4–12% Criterion^™^ XT Bis-Tris Gel (Biorad). Western blots were carried out with SHA31 antibody (1:10000), SAF32 (1:2000), actin (1:10000). Peroxidase-conjugated secondary antibodies to mouse were used (GE Healthcare) and blots were revealed with ECL 2 Western Blot detection reagent (Thermo).

### Immunofluorescence

MDCK cells, grown either on coverslips, on transwell filters, or in Matrigel^™^ were washed with phosphate-buffered saline containing CaCl_2_ and MgCl_2_ (PBS++) and then fixed. Cysts were fixed for 30 minutes at room temperature in 2% paraformaldehyde + 0,02% glutaraldehyde, while coverslips and transwell filters were fixed with 4% paraformaldehyde + 0,02% glutaraldehyde. After 15 minutes of incubation with 50 mM NH_4_Cl, 2 washes with PBS+/+ cells were saturated in non-permeabilizing buffer (PBS +/+, 20% Goat Serum) or permeabilizing buffer (0,2% Triton, 0,04% Tween 20, 20% Goat serum in PBS +/+) for 1 hour. Primary antibodies used in immunofluorescence SHA31 (1:500), SAF32 (1:200), were detected with Alexa-488 or Alexa-546 conjugated secondary antibodies (1:500). Phalloidin-Alexa647 (1:100) was used to stain actin. For 3D cultures an additional incubation with glycine (7,5 g/l in PBS +/+) for 15–30 minutes was performed before adding primary antibodies and 2 additional glycine incubations 15–30 minutes each before addition of the secondary antibodies [44]. All treatments of the 3D cysts were performed with continuous gentle agitation. Nuclei were stained with DAPI for 5 min, chambers were removed and the slides were mounted with Mowiol. The images were acquired using a laser scanning confocal microscope (LSM 700; Zeiss) equipped with a Plan Apo 63× oil immersion (NA 1.4) objective lens.

### Antibody transcytosis assay

For the transcytosis assay in 2D, cells grown on polycarbonate filters for 5 days were incubated 3h on ice with primary anti-PrP antibodies SHA31 (1:500) and SAF32 (1:200) in basolateral media. Cells were then washed 3 times with cold DMEM and incubated at 37°C for 3 hours. Cells were then fixed with 4% paraformaldehyde + 0,02% glutaraldehyde for 30 minutes at room temperature, washed 15 minutes with 50 mM NH_4_Cl and an immunofluorescence was performed with the appropriate Alexa-labeled secondary antibody, DAPI and Phalloidin-Alexa647.

For the transcytosis assay in 3D, MDCK cysts grown in Matrigel^™^ for 5–10 days were incubated for 3h or overnight at 37°C with primary anti-PrP antibodies SHA31 (1:500) and SAF32 (1:200) in the growth media. Incubation at 4°C for 3h was used as a control condition. Cysts were fixed with 2% paraformaldehyde + 0,02% glutaraldehyde for 30 minutes at room temperature, washed 15 minutes with 50 mM NH_4_Cl and the immunofluorescence was performed with appropriate Alexa-labeled secondary antibody, DAPI and Phalloidin-Alexa647.

### Colocalization assay

After fixation and immunofluorescence Z-stack images were acquired using a Zeiss LSM700 confocal microscope with a 63x oil plan apochromat objective (NA 1.4) to eliminate chromatic aberration. Colocalization analysis was performed using the Coloc 2 plugin on ImageJ software [45] (http://fiji.sc/Coloc_2).

### Biotinylation and Streptavidin Precipitation

Biotinylation was performed according to the standard protocol [46,47] with modifications. Biotinylation of monolayers on Transwell^™^ filters with s-NHS-biotin was carried out twice consecutively for 20 min at 4°C with 0.5 ml for the apical chamber and 1 ml for the basolateral chamber. Free biotin was blocked with 50 mM NH4CI in PBS containing MgCI_2_ and CaCI_2_. After washes 0.5 ml of DMEM was placed in apical and basolateral chambers and cells were incubated 3h at 37°C. Media was harvested; centrifuged 5 min 5000 rpm to remove cell debris, supernatants were supplied with 150 mM of NaCl and protease inhibitors. Media were incubated for 12 h with Streptavidin-sepharose (GE). After incubation, the beads were washed (PBS, 150mM NaCl, 0,2% BSA) 3 times consecutively (1 hour each wash) at 4°C. After the washes the beads were treated with PNG^ase^ and subjected to Western Blot.

### Statistical analysis

All graphs show the mean+/- S.E.M. from at least 3 independent experiments. Mann–Whitney U test was used to evaluate the significance of nonparametric data. Paired two-tailed t tests were used for the apical vs basolateral signal distribution *p < 0.05, ** p< 0.01

## Results

### N- and C-terminal antibodies reveal different PrP localization in 2D and 3D polarized MDCK cultures

**Fig 1:**
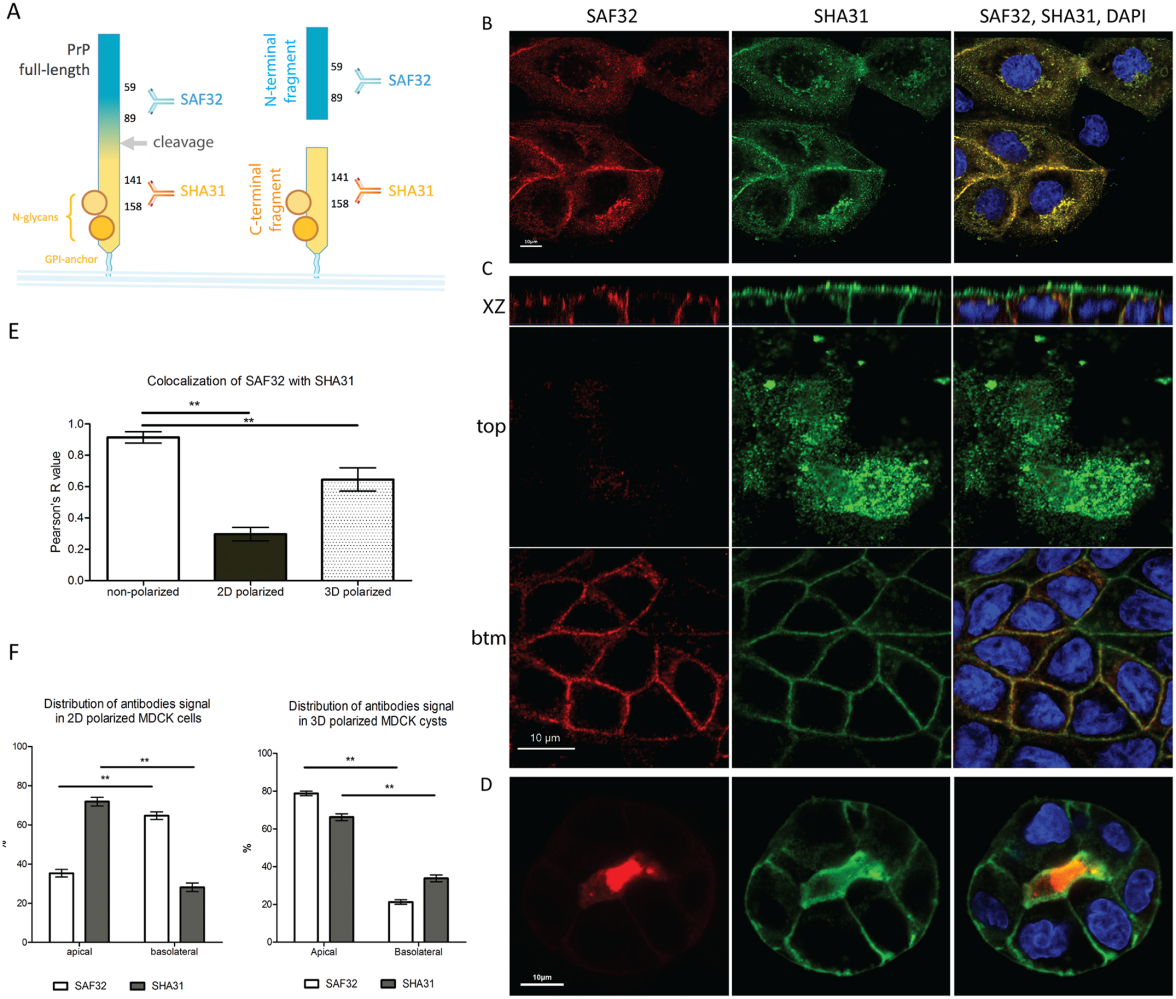
Different localization of PrP in non-polarized and polarized MDCK cells in 2D and 3D, revealed by using different antibodies. **(A)** Schematic representation of mouse PrP α-cleavage site and the recognition sites of antibodies used in this paper. The C-terminal part of PrP (orange) is membrane-attached via GPI-anchor; it has 2 independently occupied glycosylation sites (orange circles). Proteolytic α-cleavage occurs at the position 109*110. After the cleavage C-terminal fragment (C-ter) stays on the plasma membrane. C-ter as well as PrP full-length (FL) is recognized by SHA31 antibody (orange). The N-terminal part (blue), becomes a soluble N-terminal fragment (N-ter) upon cleavage. N-ter and PrP FL are recognized by SAF32 antibody (blue). **(B), (C)** and **(D)** Immunofluorescence images of MDCK cells stably expressing PrP (MDCK PrPwt cells). Non-polarized cells—plated for 24 hours on Matek (B), polarized MDCK -5 days on Transwell filters (C) 3D polarized cyst- 5 days in Matrigel^™^ (D) were fixed and immunostained for PrP using SAF32 antibody (red) and SHA31 antibody (green) and nuclei are stained with DAPI (blue). Scale bars 10 μm. In **(C)** serial confocal sections of 0,3 μm were collected from the top to the bottom of the cell monolayer. **(E)** Pearson’s R values revealing colocalization of SAF32 and SHA31 signal in non-polarized, 2D and 3D polarized states. (**p ≤ 0.01, Mann-Whitney test) **(F)** Quantification of apical vs. basolateral distribution of SAF32 and SHA31 in 2D polarized MDCK cells (left panel) and in 3D polarized MDCK cysts (right panel). These experiments were performed 3 independent times (20 cells quantified per experiment) and a total of 60 cells were used for the final quantification. (** p ≤ 0.01, paired t-test).

Polarized epithelial MDCK cells were previously used to characterize the exocytic pathway of PrP [28–31,37]. The localization and intracellular traffic of PrP in stably transfected MDCK cells is contradictory as some studies reported PrP to be apical [28,37] while others have shown PrP to be basolateral [29–31]. Since in epithelial cells protein localization depends directly on the polarity state of the cells [48] we decided to compare PrP localization in non-polarized and fully polarized MDCK cells grown on filters (hereafter referred to as a two-dimensional (2D) system), as well as in polarized 3D MDCK cysts growing in Matrigel^™^. We assessed PrP localization using 2 different antibodies to PrP: the C-terminal-targeting SHA31 (recognizing epitope 148–159) and the N-terminal-targeting SAF32 (recognizing epitope 59–89) (Fig 1A and S1 Fig). As expected, in non-polarized cells both SHA31 and SAF32 antibodies recognize the same distribution of PrP at the cell surface with an almost complete overlap of signal (Fig 1B) (Pearson’s R coefficient for SAF32/SHA31 colocalization, R = 0.9; Fig 1E). Surprisingly, in polarized cells grown on filters in 2D the SAF32 and SHA31 staining clearly segregate (Fig 1C) (Pearson’s R drops to 0.3; Fig 1E). While SAF32 antibody reveals PrP staining mostly on the basolateral surface (65±2%), confirming our earlier results [29] SHA31 antibody reveals PrP enrichment at the apical membrane (72±2%) (Fig 1F), as previously shown in similar conditions in polarized MDCK cells [37]. Interestingly, in 3D cysts, SAF32 signal concentrates in the cyst lumen while SHA31 is enriched at the apical surface (66±2%), similar to 2D culture (Fig 1D and 1E; Pearson’s R value in cysts is 0.63).

### C-terminal fragment accumulates during the establishment of monolayer polarity

The immunofluorescence data show that the apparent localization of PrP depends on the antibody used and its epitope recognition site (Fig 1A). To explain the difference in localization obtained with two different antibodies we hypothesize that PrP in MDCK cells is proteolytically processed and truncated fragments are sorted differently from PrP FL (full-length). Indeed SAF32 and SHA31 antibodies should recognize not only the full length PrP but also different cleavage products respectively (Fig 1A). The most common proteolytic processing of PrP are α and β cleavages [10,49,50]. α-cleavage has already been shown to occur in ovine PrP expressed in MDCK cells [51], generating two distinct products, the N- and C-terminal fragments. However, PrP is a heavily glycosylated protein, which in SDS PAGE migrates as several bands (1 diglycosylated, 2 monoglycosylated and 1 non-glycosylated band) [1] making biochemical distinction between PrP FL and sugar-bearing truncated fragments difficult. Therefore, to clearly distinguish full-length and cleavage fragments biochemically the different samples were treated with PNG^ase^ to deglycosylate PrP.

**Fig 2:**
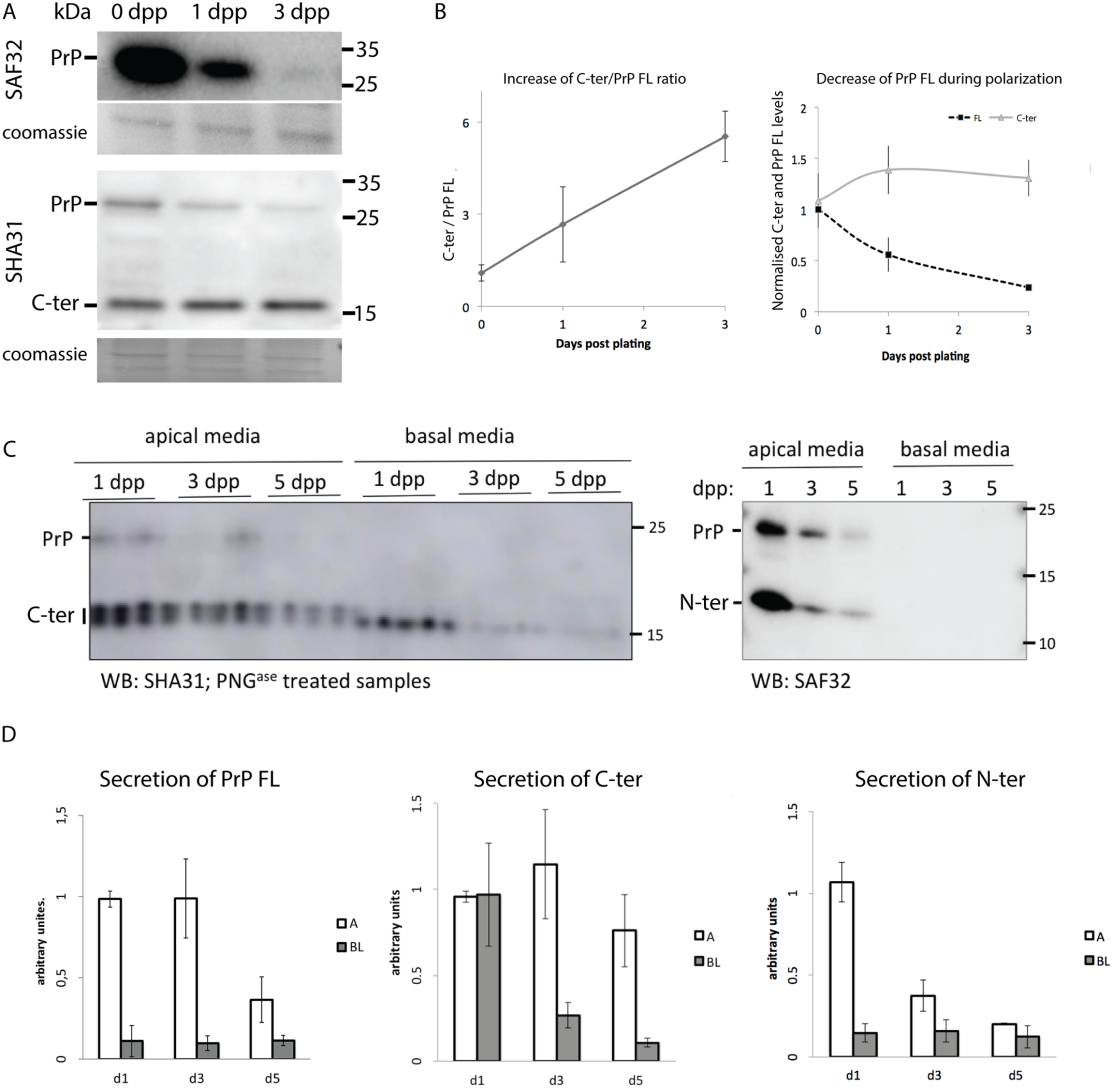
PrP cleavage through establishment of polarity in 2D MDCK cells. **(A)** MDCK PrP wt was plated in Transwell^™^ filters, then lysed at 6 hours (0 days), 1, and 3 days post plating. Cell lysates were PNG^ase^ treated and analyzed by western blot, revealed with SAF32 (upper panel) or SHA31 (lower panel) antibodies. **(B)** Left: Quantification of C-ter/PrP FL ratio through time. Right: normalization (using Coomassie blue staining) of C-ter and PrP levels with time. 4 independent experiments were quantified. **(C)** MDCK PrP wt was plated on Transwell^™^ filters for 5 days. At 1, 3 and 5 days post plating growth media was replaced by serum-free media for 3 h. This media was collected and secreted proteins were methanol precipitated from the apical or basolateral media. Precipitated media was subjected to PNG^ase^ treatment and western blotting with SHA31 antibody (left) and SAF32 antibody (right). Of note, we detect 2 different bands around 15 kDa in the apically secreted C-terminal fragment, while we detect a single band of C-ter in the basolateral media. **(D)** Quantification of PrP FL revealed with either SAF32 or SHA31 (left panel), C-ter revealed with SHA31 (middle panel) secretion and N-ter revealed with SAF32 (right panel). All densitometry quantifications were normalized to day 1. 3 independent experiments were quantified. (*p ≤ 0.05; **p ≤ 0.01, Mann-Whitney test).

To follow the presence and levels of PrP FL and its cleavage fragments during polarity establishment (Fig 2A), cells were plated on 6-well filters at a density of 2 million per well. Filters were lysed at day 0 (6 hours post plating), 1 and 3 days; cell lysates were PNG^ase^ treated and subjected to Western Blotting. Full length PrP could be detected (corresponding to a band of ~ 27 kDa) by both SAF32 antibody and SHA31 antibody (Fig 2A). SHA31 further detected a truncated PrP form around 15 kDa, which appears to correspond to C-ter fragment revealing that PrP undergoes proteolytic processing, releasing fragments that can be differentially detected by the two antibodies[8, 9, 11].

Interestingly, while in non-polarized conditions (0 dpp) PrP FL and C-terminal fragment (C-ter) are present in comparable amounts, in fully polarized cells (3 dpp) C-ter appears to be 5 times more abundant than full-length PrP (Fig 2A lower panel). The ratio of C-ter to PrP FL increases with monolayer maturation on filter (Fig 2B left). Interestingly the ratio of C-ter to the total protein load (evaluated with Coomassie blue) is stable over the polarization process, while the PrP FL level constantly decreases as shown by both the use of SAF32 and SHA31 antibodies (Fig 2A and 2B right). There are at least two hypotheses that could explain the increase of C-ter/FL ratio through polarity establishment. PrP might be subject to differential cleavage over time as polarity establishes. Alternatively, PrP FL and C-ter may have different lifetimes in non-polarized versus polarized conditions. Therefore, we next investigated whether PrP FL and C-ter fragments have a different lifetime in non-polarized and polarized conditions. To this end, non-polarized and polarized MDCK cells were incubated in absence or in presence of a protein synthesis inhibitor cycloheximide (CHX) for 1, 4 and 6h. Cell lysates were PNG^ase^ treated, and subjected to Western Blotting with SHA31 antibody. In non-polarized MDCK cells, PrP FL was not detectable after 4h of incubation with CHX while C-ter is decreased but still detectable after 6h of incubation. We could estimate that in non-polarized conditions, the lifetime of PrP FL is less than 2h and that of C-ter is around 4.5 hours. Interestingly in polarized conditions, we calculated that the estimated lifetime of PrP FL is roughly 90 minutes while that of C-ter is around 5.5 hours, indicating that the lifetime of C-ter is longer in polarized MDCK cells compared to non-polarized conditions (S2 Fig) and that C-ter lifetime is longer than FL lifetime in both conditions.

### N-terminal fragment is apically secreted

As the C-terminal fragment is a membrane-bound and relatively stable fragment (S2 Fig) [8,10] in order to understand if PrP cleavage increases along with the monolayer maturation process, we decided to monitor the kinetics of secretion of the soluble N-terminal (N-ter) fragment that directly reflects the occurrence of the cleavage process. To this end we analyzed PrP secretion in the filter culture medium during the establishment of the polarized monolayer. 1, 3, and 5 days post plating serum-free media was added to the cells for 3h, then the conditioned media was harvested for analysis. Proteins were methanol-precipitated from the media and loaded on the gel or subjected to PNG^ase^ treatment, western blot and revealed with SHA31 or SAF32 (Fig 2C). We found that PrP FL and soluble N-ter are secreted exclusively in the apical media throughout the process of monolayer maturation (Fig 2C right and Fig 2D left and right). Interestingly, the greatest amount of N-ter secretion is at 1 day post plating (Fig 2D right). This might indicate that PrP cleavage is most active in non-polarized conditions or in the beginning of polarity program activation. Alternatively, if the cleavage occurs only at the apical surface one explanation is that more PrP goes directly to the forming apical surface in early polarity stages rather than in late polarity stages, as a possible result of PrP trafficking changes during the establishment of the polarized phenotype. Consistent with apical cleavage in polarized cells (3 dpp and 5 dpp) C-ter is secreted in larger amounts into the apical media than into the basolateral one (Fig 2C left and Fig 2D center). Basolateral secretion of C-ter occurs mainly at 1 dpp, and then it decreases dramatically (Fig 2D center), in agreement with the apical enrichment of C-ter during polarity establishment of the MDCK monolayer (Fig 1C and S1 Fig).

**Fig 3:**
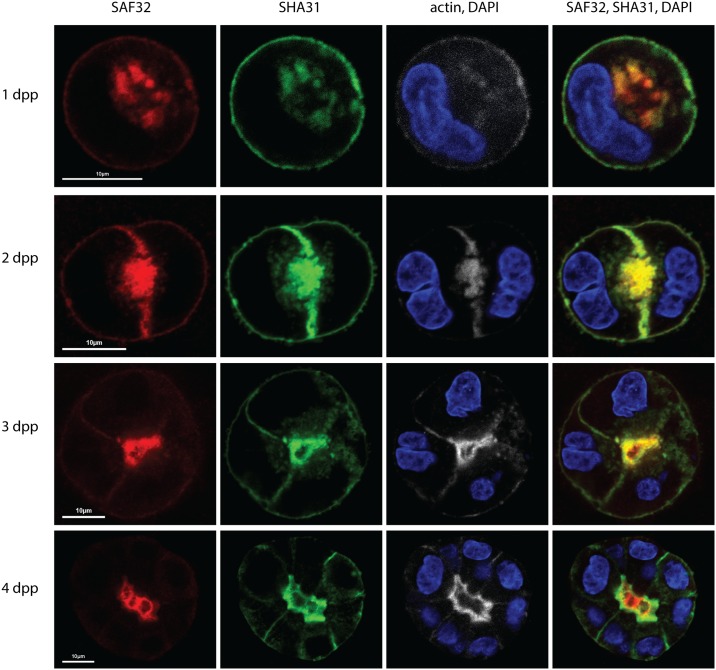
PrP distribution changes upon cyst maturation. MDCK PrP wt cells were plated in 2% Matrigel^™^, and then fixed at 1 day (1 dpp), 2 days (2 dpp), 3 days (3 dpp) and 4 days (4 dpp) post plating. Cysts were co-stained with SAF32 (red), SHA31 (green), phalloidin-Alexa-647 (white) and DAPI (blue). Scale bar 10 μm. Representative images are shown from a total of 3 experiments.

We then characterized the dynamics of PrP cleavage fragment, localization and secretion in 3D culture during cyst maturation. We analyzed PrP localization by immunofluorescence using SHA31 and SAF32 antibodies on 1, 2, 3 and 4 day-old cysts (Fig 3). At early time points (1 and 2dpp) SAF32 and SHA31 colocalize (R = 0,86 ± 0,06). At the earliest stage of 1 dpp, when the apical membrane is not yet established SAF32 and SHA31 distribution is similar to the non-polarized cells growing on coverslips. As soon as membrane polarity is established, PrP is enriched on the apical membrane as revealed by both antibodies. In the 4-day old cysts however, SAF32 and SHA31 signals segregate from each other, (Pearson’s R decreases up to 0,67 ± 0,1). Specifically, we observed different dynamics for the two antibodies. Figs 1D and 3 demonstrate that SAF32 signal is mainly luminal at later stages of cyst maturation (4 and 5 dpp). However, at day 2, an early stage of cyst development, we could detect staining on the basolateral surface, which progressively disappeared with time until day 4 when the entire signal was observed in the cyst lumen. On the contrary, SHA31 distribution does not significantly change from day 2 to day 4, as it is enriched at the apical membrane from day 2 and its basolateral staining is stable with time (Fig 3).

### PrP undergoes basolateral-to-apical transcytosis in fully polarized MDCK cells

The progressive enrichment of SAF32 signal in the apical lumen concomitant with its progressive disappearance from the basolateral membrane prompted us to investigate whether PrP undergoes basolateral to apical transcytosis that progressively increases with maturation of the epithelium.

**Fig 4:**
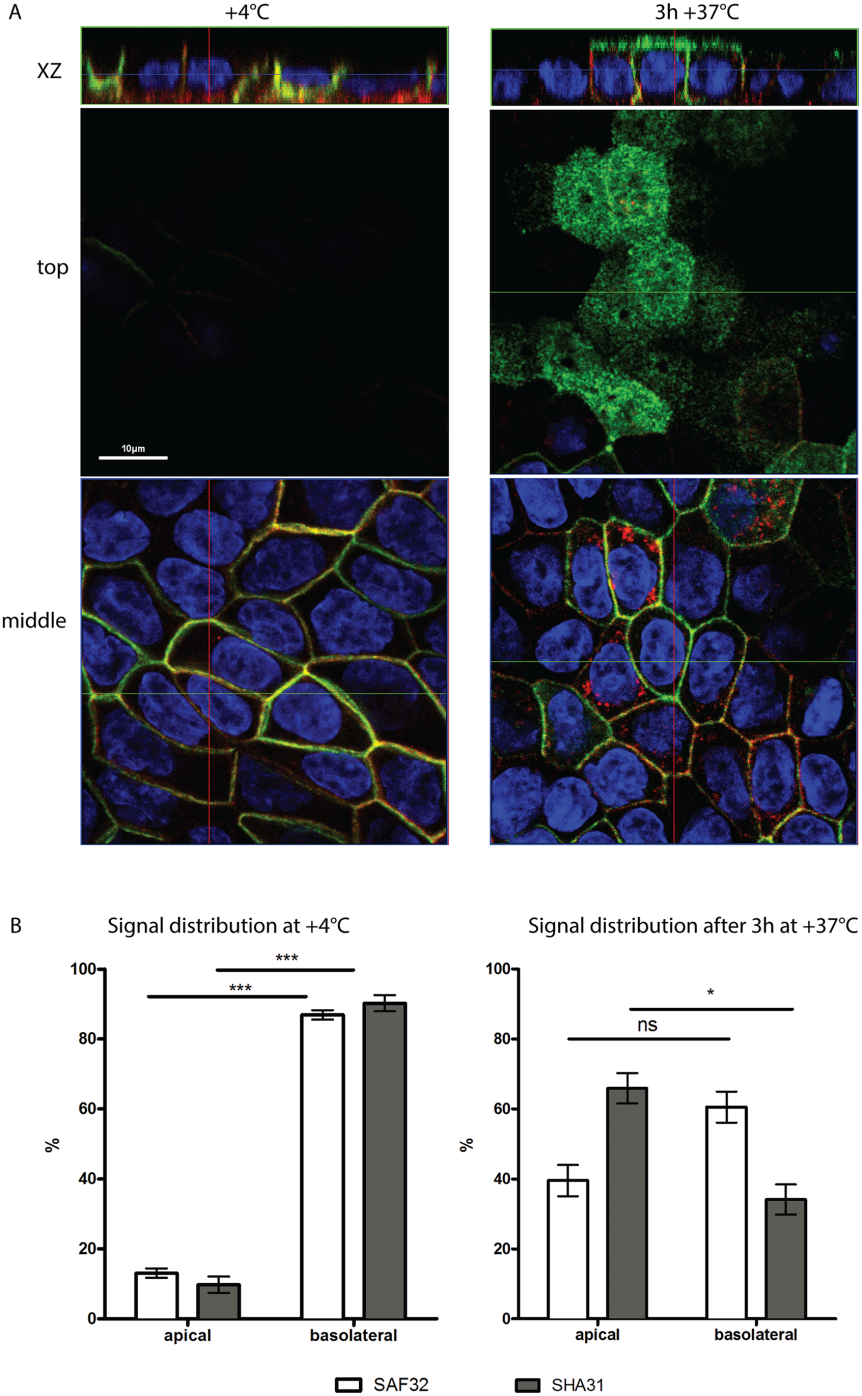
PrP wt undergoes basolateral to apical transcytosis in 2D polarized MDCK. **(A)**. After 5 days of polarization MDCK cells were incubated with SHA31 (green) and SAF32 (red) in the basal media for 2 hours on ice, then filters were washed and incubated for +4°C as a control (left column) or for 3h at 37°C (right column). After fixation cells were permeabilized and stained with secondary antibodies and DAPI (blue). **(B)**. Quantification of the apical vs basolateral antibody distribution for 3h of incubation at 4°C (left) or at 37°C (right). Note that after the 3h of incubation SAF32 is present in intracellular sub-apical vesicles contributing to the quantification. The experiment was repeated 5 times, and a total of 100 cells were included in the analysis. (*p ≤ 0.05; ***p ≤ 0.001 Paired t test).

To sustain this hypothesis, we directly investigated the occurrence of basolateral-to-apical transcytosis in MDCK cells fully polarized in 2D and in 3D. To this aim, we first performed an antibody-based transcytosis assay on MDCK cells grown on filters (Fig 4). Polarized monolayers grown on filters for 5 days were incubated with SHA31 and SAF32 antibodies in the basolateral chamber for 2 hours at +4°C (to saturate basolateral PrP with antibodies). Then filters were washed to remove unbound antibody and placed for 3h at +4°C (to prevent antibody internalization and transcytosis) or at +37°C. After fixation, cells were permeabilized and stained using fluorescently labelled secondary antibodies, and DAPI. As shown in Fig 4 at +4°C both SHA31 and SAF32 staining are restricted to the basolateral membrane (Fig 4B left). After the incubation at 37°C SHA31 signal is significantly enriched on the apical membrane, (Fig 4B right, 66±4% of SHA31 is detected at the apical surface), indicating occurrence of transcytosis. We did not observe a similar apical signal for SAF32. Instead after 3 hours of incubation with SAF32 antibody at 37°C, SAF32 signal on the basolateral membrane becomes weaker compared to the signal at +4°C and is abundantly revealed in intracellular vesicles (S3 Fig). These results are consistent with antibody internalization from the basolateral membrane and with the cleavage occurring on the way to the apical surface or at the apical surface, releasing N-ter into the apical medium, thereby explaining why SAF32 signal is undetectable on the apical surface.

**Fig 5:**
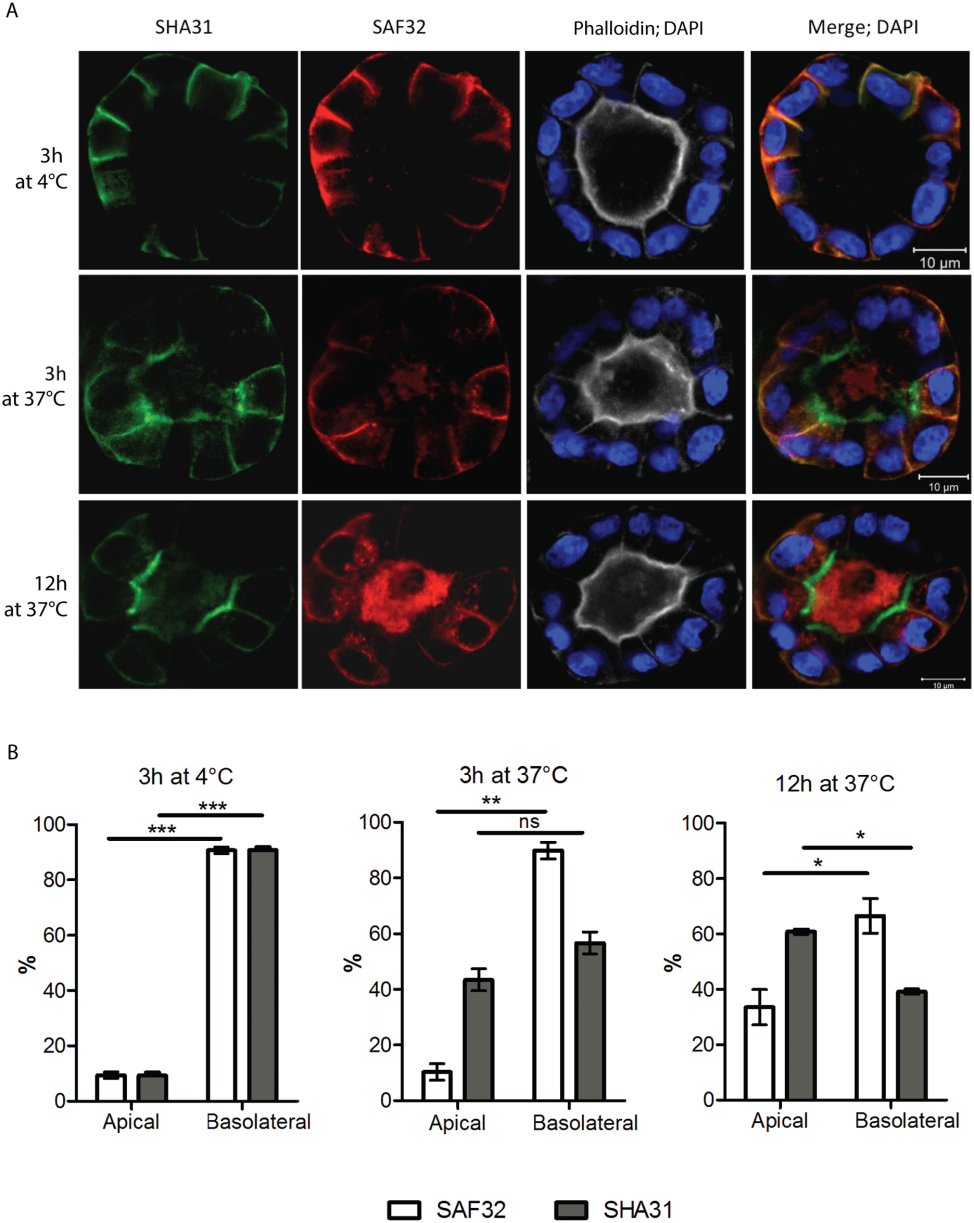
PrP wt undergoes basolateral to apical transcytosis in 3D polarized MDCK cysts. **(A)** Fully polarized, mature cysts were incubated with the mix of SHA31 (green) and SAF32 (red) antibodies for the indicated time. Incubation at +4°C was used as a control. After fixation, cysts were permeabilized and stained with secondary antibodies, phalloidin-Alexa647 (gray) and DAPI (blue). **(B)** Quantification of the apical vs basolateral antibody distribution in case of 3h of incubation at 4°C (left), 3h at 37°C (center) and overnight at 37°C (right). Note that starting at 3h and especially after an overnight incubation, SAF32 is present in intracellular vesicles and in the lumen, contributing to the quantification. The experiment was repeated 3 times, and a total of 120 cells were included in the analysis. (*p ≤ 0.05; **p ≤ 0.01; *** p ≤ 0.001 Paired t test).

To confirm these results, we investigated the occurrence of PrP transcytosis in fully polarized cysts in 3D culture. 5 to 10 days polarized cysts were incubated with the mix of SHA31 and SAF32 antibodies at 37°C for different times, while incubation at +4°C for 3h was used as a control. After fixation cysts were permeabilized and the localization of these antibodies visualized with respect to the apical and basolateral membranes (Fig 5). We found that both antibodies were able to bind the basolateral surface, and progressively transcytose towards the apical membrane. However, while SHA31 signal gradually enriches the apical rim of the apical lumen, SAF32 fluorescence is found mostly in the lumen where it accumulates with time (over-night incubation). Interestingly, during the transcytosis experiment we do not detect any SHA31 antibody signal in the lumen, suggesting that there is no or very little secretion of PrP FL or C-ter in the lumen. Upon transcytosis, PrP is cleaved and membrane-bound C-ter fragments remains at the apical surface, while N-ter is secreted into the lumen. These combined experiments show clearly that PrP undergoes basolateral-to-apical transcytosis and indicate that cleavage occurs upon arrival to the apical membrane.

**Fig 6:**
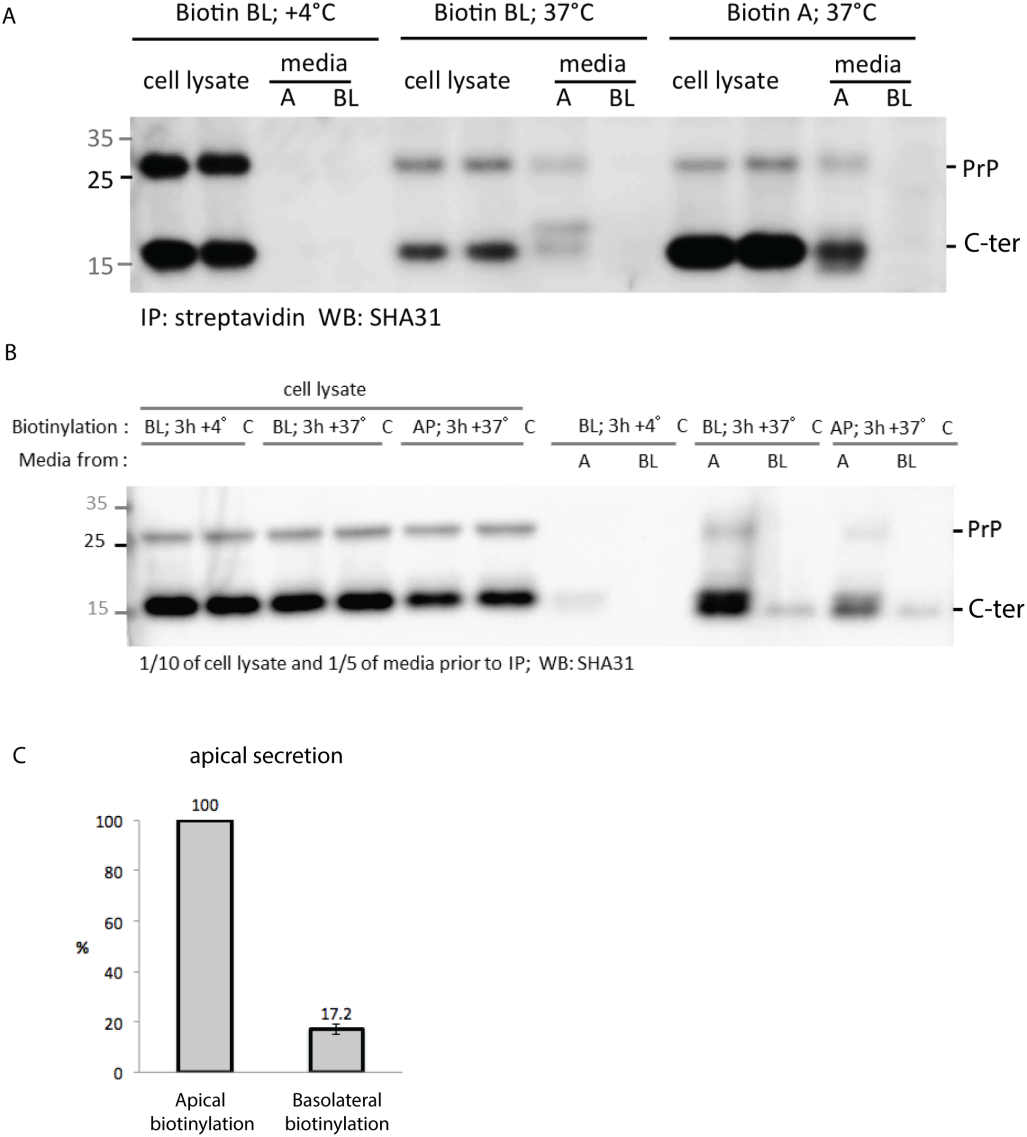
PrP follows a transcytotic route at steady state. **(A)** After 5 days of polarization MDCK cells were biotinylated on the basolateral or apical membrane. After surface biotinylation cells were allowed to secrete in serum-free media for 3 hours. Media was collected, streptavidin-precipitated, PNG^ase^ treated and then analyzed by western blot using SHA31 antibody. **(B)** 1/10 of cell lysate and 1/5 of media before the streptavidin-precipitation was deglycosylated and run on western blot. **(C)** Transcytosis quantification. The relative amount of PrP, biotinylated on the basolateral membrane and streptavidin-precipitated in the apical membrane was normalized to the amount of PrP biotinylated on the apical membrane and precipitated from the apical media. Quantification was done on 4 independent experiments.

To rule out the possibility that antibody binding induces the observed transcytosis and to confirm that PrP undergoes transcytosis in physiological conditions (i.e., in the absence of antibody) we used a biotinylation assay [52]. MDCK PrP wt cells were fully polarized on filters for 5 days and the basolateral membrane was biotinylated on ice. After washing to remove unbound material, serum-free culture media was added and filters were placed at +4°C (as a control) or at 37°C for the transcytosis assay. At the end of 3h apical and basolateral media were recovered and biotinylated proteins were precipitated from the media by incubation with immobilized streptavidin. Recovered proteins were deglycosylated, run on SDS-PAGE and revealed by western blot with SHA31 antibody (Fig 6). We observed that PrP initially residing in basolateral membrane could be detected in the apical media. This is likely due to transcytosis of the biotinylated PrP, as in control experiments where we incubated the filters at +4°C instead of 37°C, secretion of PrP was dramatically decreased (Fig 6A). Additionally, all the PrP that was apically secreted at +4°C was not biotinylated (Fig 6B), confirming that apical secretion of PrP biotinylated on the basal membrane is an active process. We compared the amount of transcytosed and secreted PrP, using as a positive control the secretion of PrP after apical biotinylation. We found that 17±4% of secreted PrP comes from the basolateral membrane (Fig 6C). This data combined with the data from the antibody transcytosis assays confirms that PrP undergoes transcytosis in steady state conditions.

## Discussion

In the present work, we used both C-terminal (SHA31) and N-terminal (SAF32) antibodies to study PrP localization and intracellular trafficking in fully polarized MDCK cells. We observed that in polarized MDCK cells SAF32, which recognizes PrP FL and the soluble N-terminal fragment, stains intracellular vesicles (S3 Fig) and the basolateral membrane in agreement with our and other previous findings [29–31]. On the other hand when using the SHA31 antibody, which recognizes PrP FL and membrane-anchored C-terminal fragment we obtained a clear apical signal for PrP, as shown before by Christensen and Harris [37]. Apical SHA31 signal is most likely due to recognition of C-ter, which is not recognized by SAF32 antibody. We found that C-ter segregates from PrP FL both in 2D and in 3D polarized cells. An advantage of 3D culture is the presence of the isolated apical lumen that is physically separated from the basolateral media. Using SAF32 antibody we could detect a strong luminal signal that most probably results from luminally trapped soluble N-ter, although we cannot exclude that N-ter is capable of membrane binding, as previously reported [53–56]. Thus by combining the use of the two different antibodies our data reconcile and explain the conflictual results in the literature on PrP localization in MDCK cells.

We next used a biochemical approach to study PrP cleavage in MDCK cells (Fig 2). C-terminal fragment is present both in non-polarized and polarized MDCK cells; interestingly, in polarized cells C-ter is 5 times more abundant than PrP FL. At least two scenarios could explain the increase of C-ter/PrP ratio during the establishment of epithelial polarity; either an increase of PrP cleavage though polarity maturation and/or a difference in the lifetime of PrP FL and C-ter in polarized versus non polarized cells. Our results indicate that the lifetime of C-ter is longer in polarized versus non-polarized conditions (S2 Fig). However, by assessing the presence of cleavage fragments in the media, we could show that secretion of PrP and its cleavage fragments is polarized: PrP FL, N-ter and C-ter are secreted apically while only C-ter is found in the basolateral media. Basolateral secretion of C-ter significantly decreases during monolayer maturation and this could result from an increase of the efficiency of protein sorting during polarity establishment in correlation with a decrease of missorting protein [57,58]. This hypothesis also implies that C-ter bears a potent apical sorting signal. Nevertheless our data show that there is a decrease of N-ter secretion in the apical medium (Fig 2C right panel and Fig 2D right panel); C-ter stability in polarized cells is higher than in non-polarized MDCK cells (S2 Fig) and PrP transcytoses from the BL to the AP surface (Figs 4 and 5).

Collectively, our data strongly indicate that the fraction of basolateral-sorted PrP is redirected towards the apical surface thereby explaining the decrease of basolaterally secreted C-ter. Furthermore our findings suggest that while apically sorted PrP is secreted as full length protein into the apical media, it can also undergo cleavage, releasing the fragments. This is consistent with, and explains, our previous data [29] showing that newly synthesized PrP is sorted to both apical and basolateral surfaces, but then, while the signal was stable on the basolateral membrane, it quickly disappeared from the apical surface [29]. This is sustained and explained by the fact that while the N-terminal antibody does not recognize the protein on the apical domain, this latter is recognized by the C terminal antibody (reacting against the C-terminal cleaved fragment on the apical surface). Consistently, the fact that SHA31 reveals high signal at the apical surface (Fig 1C and 1D and S1 Fig) indicates that there is an enrichment of C-ter on the apical membrane over the full-length, a situation reinforced by the longer lifetime of C-ter in polarized cells.

Interestingly we found that while in the apical media there were 2 forms of a C-terminal fragment previously described in BHK cells as “membrane-anchored” and “devoid-of-GPI anchor” C1 [59], in the basolateral media we revealed only one form of C-terminal fragment. It seems probable that, in MDCK cells as well as in BHK [59] there are at least 2 mechanisms of shedding and release of PrP into the medium. The unidirectional secretion of PrP and several cleavage fragments in MDCK indicates that PrP cleavage in our model occurs either on the apical membrane or in an intracellular compartment on the way to the apical surface; this is also in agreement with data showing that GFP-PrP and its cleavage fragments are secreted from the apical side of polarized MDCK [51].

Next, we investigated the dynamics of the secretion during the polarity establishment. We observed that in cell lysate C-ter/PrP FL ratio gradually increases throughout polarization (Fig 2D). Because the secretion of soluble N-terminal fragment is maximal at early stages of polarization it is not likely that cleavage increases throughout the process of polarization. Rather, it appears that the increasing ratio is due to a progressive decrease of PrP FL upon polarization coupled with the greater stability of C-ter (Fig 2A and S2 Fig). In support of this hypothesis, an immunofluorescence of the early polarization stage reveals significant apical signal of PrP full-length detected by SAF32 antibody (S1 Fig). Further experiments will be required to investigate this mechanism.

PrP full-length is basolateral and C-terminal fragment is apical, but how is this steady state distribution maintained? We previously demonstrated through pulse-chase experiments that PrP-FL reaches the apical and basolateral membranes simultaneously (40/60 respectively). Interestingly, we further revealed that the protein was unstable on the apical surface [29]. The results presented here allowed us to hypothesize that the re-localizion of PrP FL to the apical membrane is due to transcytosis. As PrP is a non-conventional GPI-AP, exhibiting features different from other GPI-APs [60], we investigated if PrP takes a transcytotic route in polarized MDCK cells. We found that in both 2D and 3D cultures of polarized MDCK cells PrP efficiently undergoes basolateral-to-apical transcytosis. To monitor transcytosis we have used N-terminal and C-terminal antibodies that can cluster PrP, therefore mimicking a ligand binding. In these conditions, transcytosis of PrP is very efficient: at the end of 3 hours C-terminal antibody SHA31 from the basolateral membrane is significantly enriched in the apical membrane (Fig 4B). When we monitor transcytosis with SAF32 we did not observe any significant apical enrichment, however we observe it enriched in intracellular vesicles. We hypothesize that this may be because PrP cleavage occurs, releasing the N-terminal fragment (containing the SA32 epitope) by secretion from the apical surface. Consistent with this hypothesis we can clearly see apical secretion of SAF32 in 3D polarized cells (Fig 5); thus, most likely SAF32 luminal signal is due to the proteolytic removal of the epitope from the cell surface. Our data are consistent with PrP full-length being endocytosed from the basolateral membrane and undergoing cleavage either inside the intracellular vesicular compartment on its way to the apical surface or at the apical surface. Here we did not investigate the cellular localization of post-translational processing of PrP that could occur either on its way to the apical surface or at the apical surface. However, a previous study suggested that PrP is most likely cleaved in the intracellular secretory compartment [61]. Another intriguing question is as to whether ligand binding stimulates PrP transcytosis, making PrP behave like IgA receptor in MDCK cells [62]. A recent article by Pflanzner and colleagues has shown that amyloid-β (1–40) transcytosis through the blood-brain barrier depends on PrP [63]. Here, using antibody binding, we show that PrP itself undergoes transcytosis; therefore PrP could potentially play the role of shuttle for its ligands and PrP-interacting molecules.

**Fig 7:**
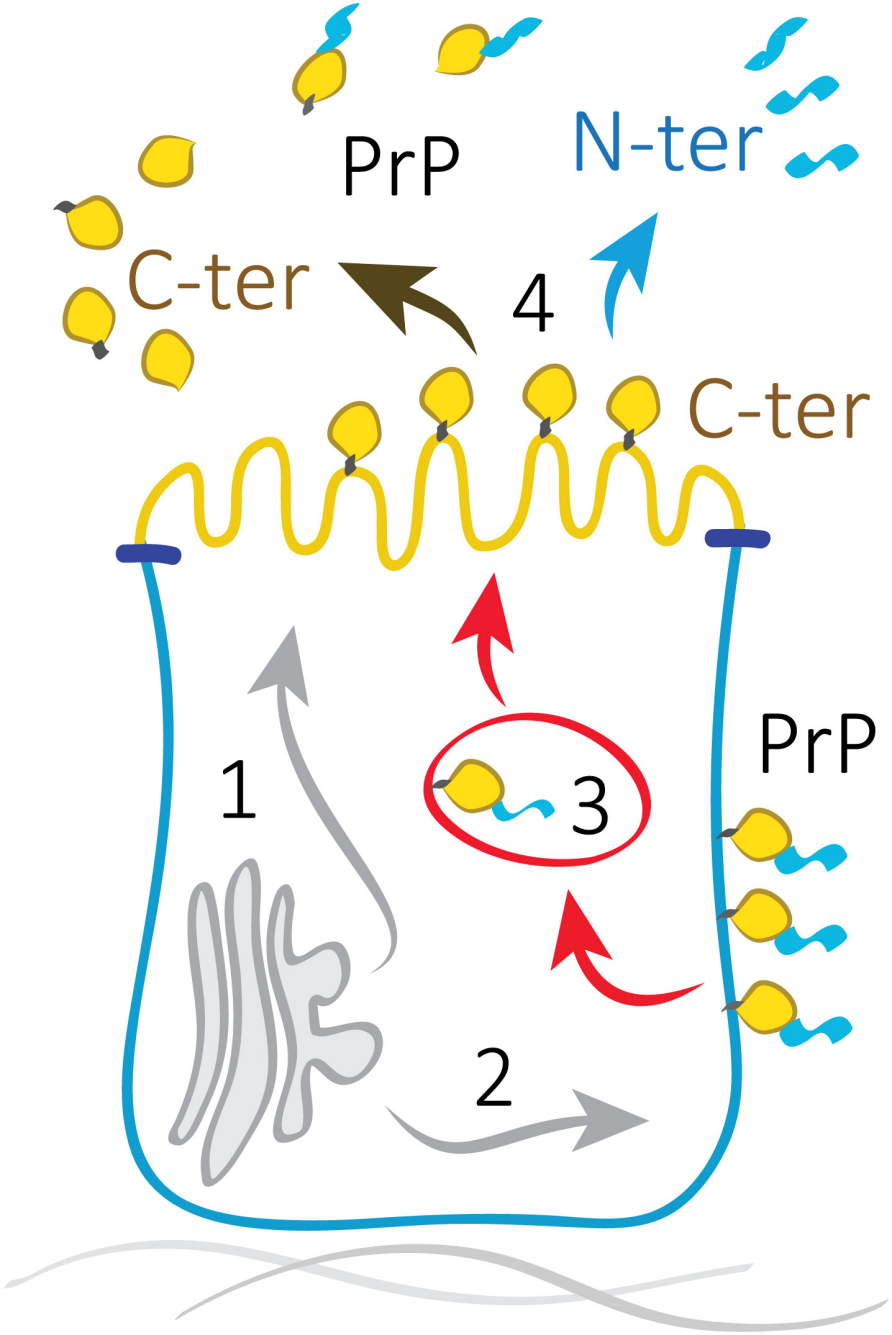
Model of PrP trafficking in MDCK cells. In fully polarized epithelial cells PrP is exported from the Golgi to both apical and basolateral surfaces (40/60) [29].**(1)** A significant part of PrP sorted from TGN to the apical membrane undergoes cleavage in the vesicular compartment or at the apical cell surface. (2) PrP sorted from TGN to the basolateral membrane stays intact. **(3)** Basolateral PrP is transcytosed and a part of it is cleaved on the way to the apical membrane. **(4)** Soluble N-terminal fragment is released to the apical media; full-length PrP as well as C-terminal fragment are shed from the apical media.

Our work showed that PrP in polarized cells is cleaved on its way to the apical membrane or at the apical membrane, and that it undergoes basal-to-apical transcytosis. To summarize the data of PrP in polarized MDCK cells we propose the following model (Fig 7): first, a similar amount of PrP is sorted in TGN to apical and basolateral membranes; 1. During the traffic to the apical surface a fraction of PrP molecules undergoes cleavage; 2. Basolaterally sorted PrP reaches the cell membrane intact; 3. PrP is transcytosed from the basolateral to the apical membrane where part of PrP molecules are cleaved; 4. PrP FL reaching the apical surface is shed to the media, soluble N-terminal fragment is secreted to the apical media, the C-terminal fragment is stabilized on the apical surface, and part of it is shed to the apical media.

Our findings are of fundamental importance for the GPI-AP trafficking in polarized cells. Further studies of PrP trafficking (eg., by using deletion mutants) could identify signal sequences in PrP responsible for the transcytosis and allow for a better characterization of the underlying mechanisms. By shedding some light on the polarized trafficking of PrP and its cleavage fragments, this study could help to explain the role of cleavage fragments in PrP^Sc^ formation, as for example in the case of the dominant negative effect observed for C1 fragment in this process [13].

## Supporting Information

S1 Fig Localization of PrP in early stage of polarization in 2D. Immunofluorescent pictures of MDCK PrPwt cells plated for 24 hours on Transwell filters. Cells were fixed and immunostained for PrP using SAF32 antibody (left column) and SHA31 antibody (middle column) and nuclei are stained with DAPI (right column). Scale bars 10 μm. Serial confocal sections of 0,3 μm were collected from the top to the bottom of the cell monolayer.
(TIF)

S2 Fig Kinetic of PrP degradation in polarized and non-polarized MDCK cells. MDCK PrP wt was plated sparsely either for 1 day **(A)** in 6 well plate or for 5 days at 2 million/filter in Transwell^™^ filters **(B)**, then treated with 150 μM cycloheximide and lysed after 0, 1, 4 and 6 hours of treatment. Cell lysates were PNG^ase^ treated and analyzed by western blot, revealed with SHA31 antibody. Quantifications of PrP FL and C-terminal fragment normalized to actin through cycloheximide treatment are shown on the right of the panel. 3 independent experiments were quantified.
(TIF)

S3 Fig Intracellular SAF32 vesicles. Transcytosis assay from Fig 4 (transcytosis experiment in 2D). A high magnification of a single cell after 3h of transcytosis, red arrows mark SAF32 intracellular vesicles that localize subapically and therefore contribute to the apical signal quantification.
(TIF)
