# Supplementary figures and images for: PrPC Undergoes Basal to Apical Transcytosis in Polarized Epithelial MDCK Cells
Source: PLoS One. 2016 Jul 7;11(7):e0157991. doi: 10.1371/journal.pone.0157991 (PMC4936696; doi:10.1371/journal.pone.0157991)

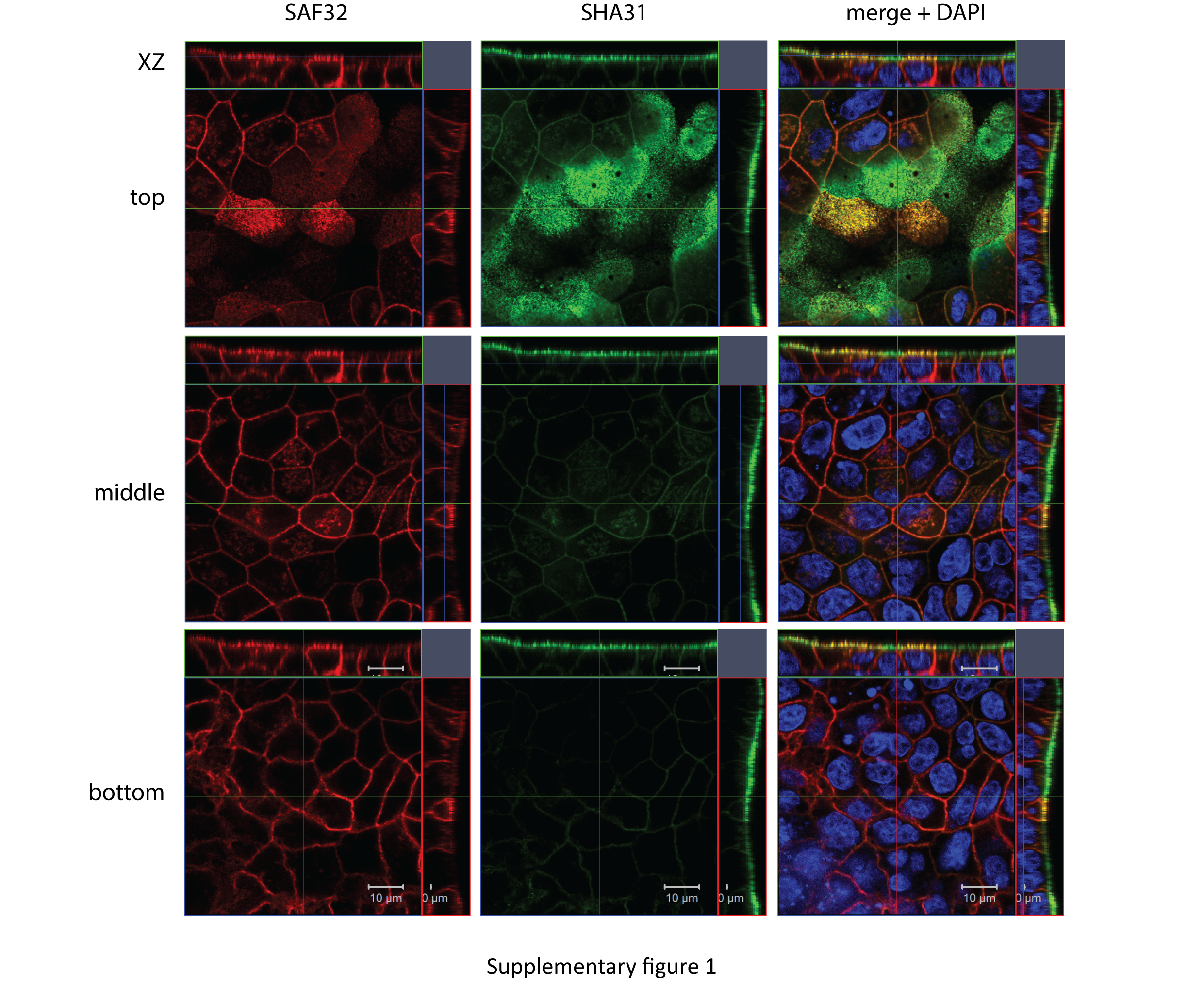

Supplement: S1 Fig — Immunofluorescent pictures of MDCK PrPwt cells plated for 24 hours on Transwell filters. Cells were fixed and immunostained for PrP using SAF32 antibody (left column) and SHA31 antibody (middle column) and nuclei are stained with DAPI (right column). Scale bars 10 μm. Serial confocal sections of 0,3 μm were collected from the top to the bottom of the cell monolayer. (TIF) [file pone.0157991.s001.tif]

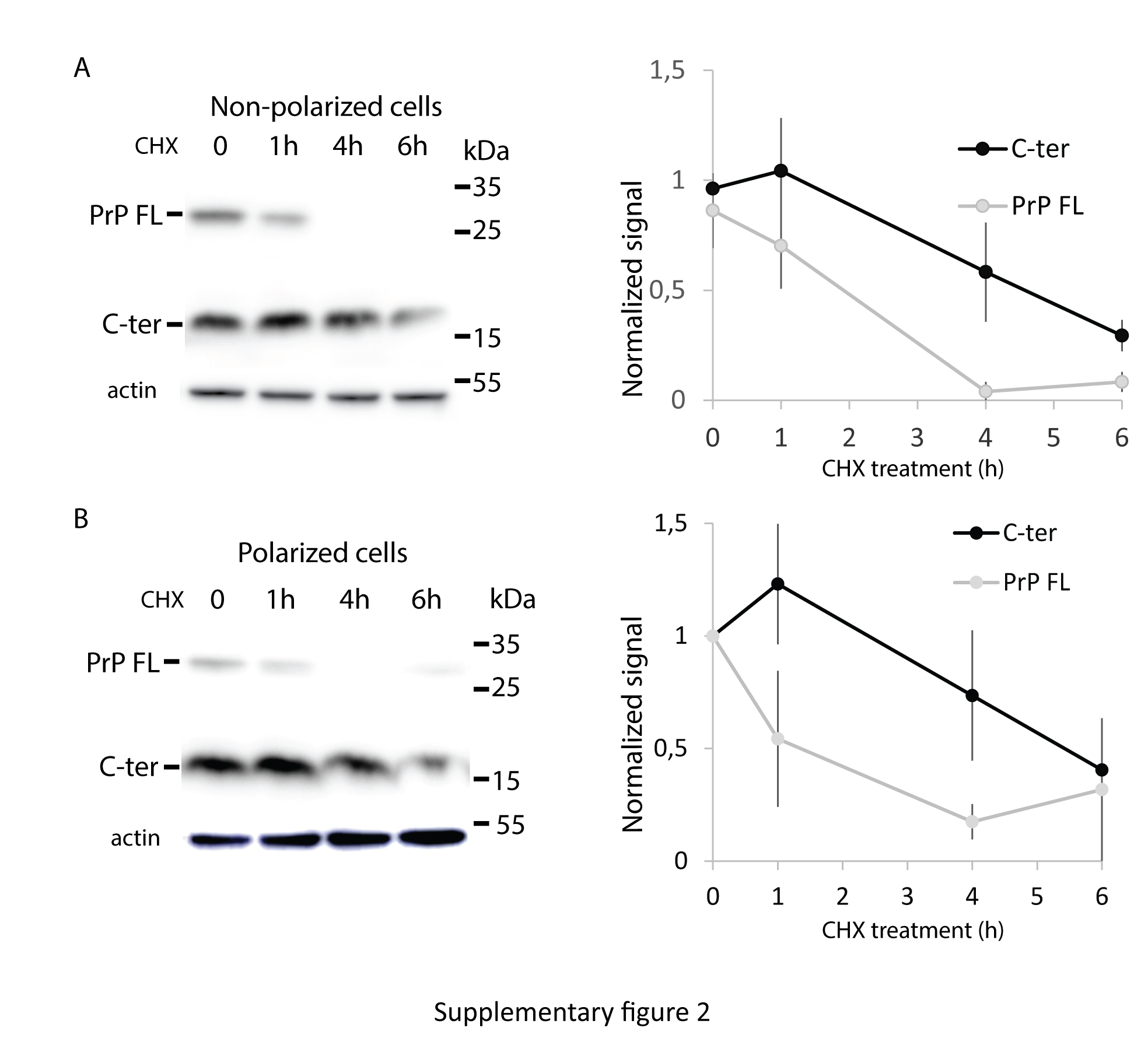

Supplement: S2 Fig — MDCK PrP wt was plated sparsely either for 1 day (A) in 6 well plate or for 5 days at 2 million/filter in Transwell™ filters (B), then treated with 150 μM cycloheximide and lysed after 0, 1, 4 and 6 hours of treatment. Cell lysates were PNGase treated and analyzed by western blot, revealed with SHA31 antibody. Quantifications of PrP FL and C-terminal fragment normalized to actin through cycloheximide treatment are shown on the right of the panel. 3 independent experiments were quantified. (TIF) [file pone.0157991.s002.tif]

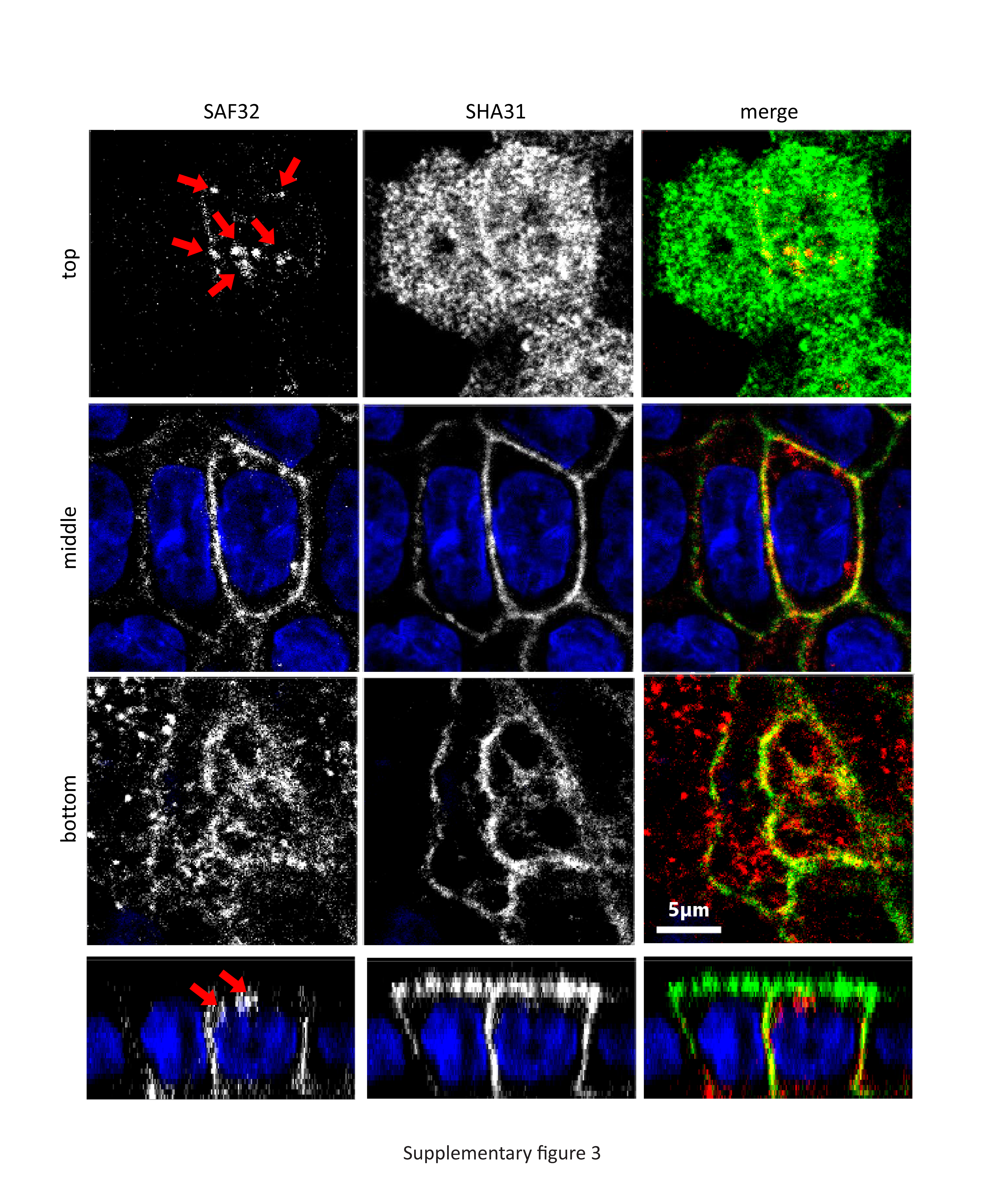

Supplement: S3 Fig — Transcytosis assay from Fig 4 (transcytosis experiment in 2D). A high magnification of a single cell after 3h of transcytosis, red arrows mark SAF32 intracellular vesicles that localize subapically and therefore contribute to the apical signal quantification. (TIF) [file pone.0157991.s003.tif]
